# Current college graduates’ employability factors based on university graduates in Shaanxi Province, China

**DOI:** 10.3389/fpsyg.2022.1042243

**Published:** 2023-01-09

**Authors:** Lijuan Jiang, Zirou Chen, Changkui Lei

**Affiliations:** ^1^Faculty of Business and Economics, University of Pécs, Pécs, Hungary; ^2^School of Safety and Emergency Management Engineering, Taiyuan University of Technology, Taiyuan, Shanxi, China

**Keywords:** employability, college graduates, employees, professional ability, factor analysis

## Abstract

With the development of higher education, there are a large number of college graduates turn into the labor market, and college students’ employability has become a popular topic. In order to explore the factors that affect employability’s improvement and what factors employability includes, this article summarizes the previous research on employability, uses university graduates of Shaanxi Province as research examples, and investigates the employability factors of college graduates. With the help of SPSS software, data analysis is conducted on the 220 valid questionnaires. The study uses reliability and validity analysis to verify the quality of the questionnaire, takes the exploratory factor analysis to test the employability factors of college students, and employs multiple linear regression analysis to test the factors that influence employability’s improvement. The results of the research show that individual traits, social experience, and workplace training have a significant impact on college students’ employability; knowledge understanding and learning ability, self-management ability, emotional intelligence, generic skills, professional ability, and career planning capability are the important factors of the employability which college students should master. Our research results update the influencing factors of employability, so that contemporary college students have a new understanding of employability, and help them to improve their employability more pertinently.

## Introduction

**FIGURE 1:**
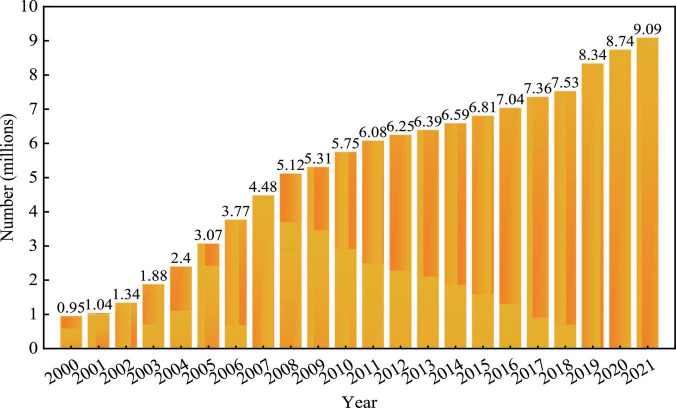
Chinese college graduates from 2000 to 2021.

In recent years, the employment environment in China is facing great challenges. Internally, the scale and number of college graduates are growing fast, the labor market is more crowded. The statistic shows that in 2021, there are 9.09 million fresh college graduates in China, shown in Figure 1, which represents an increase of 0.35 million students compared to that 2020 (National Bureau of Statistics, 2021). According to the “*2021 China College graduates employment report*” published by the recruitment company ZhiLian, a leading human resource service company, as of August 2021, 66% of college graduates of 2021 are still looking for a job (Lian and Pin, 2021). Externally, the tensioning relationship between China and US, as well as the COVID-19 pandemic, have slowed down China’s economic growth and have influenced the Chinese labor market. Furthermore, business organizations are more interested in hiring people with capabilities that cannot be easily replaced by technology, they have higher requirements and expectations for the employees’ abilities, and employees with weak working abilities will be easily eliminated by the market (Nelissen et al., 2017). As the key to the labor market, employability is the main factor to improve the functioning of the labor market, achieve full employment and develop new career models in the current economic environment (De Cuyper et al., 2011).

According to some scholars, the employability of college students with practical ability as the core is the most important factor affecting the quality of the labor market (López and Pérez, 2014), and college students are one of the main groups that need to improve their employability (Murangi et al., 2022). From the existing literature, there are differences in the focus of research in China and abroad. Foreign scholars’ studies on employability mainly focus on industrial workers or general workers, and there are few direct research results on college students. While Chinese studies on employability mainly focus on college students, however, these researches are mainly based on foreign countries’ employability research results, or from perceptual intuitive work experience, the lack of theoretical consensus on employment (Guo, 2017), and these research results are not really understood and applied to school training and education (Narula and Aithal, 2018). It led to college graduates do not know what influenced their employability and which employability they need to master; the university does not systematically and pertinently carry out employability training for students. Furthermore, modern society changes so fast, the requirements and contents of the employability of enterprises are constantly changing and updating, and students still lack full comprehension of employability (Chan, 2015). Thus, it is particularly important to investigate the employability that the new employment form requires and keep college students’ understanding of employability up to date and future trends. Studying the deviation between college graduates’ employability and social demand is not only closely related to the graduates themselves but also related to the construction of colleges in China and the long-term development of enterprises, it meets the requirements of social development (Pinto and He, 2017).

In order to investigate the systematic and comprehensive employability of college students, this paper combines domestic and foreign literature research, uses the empirical analysis method, takes Shaanxi Province as a research project, and explores and updates the factors and structure of current college graduates’ employability. Shaanxi Province locates in central China, has higher education and a large number of universities, and Shaanxi Province makes lots of efforts to increase the employment rate of college graduates. In addition to various stable employment policies for graduates, the province also makes a large number of policy guarantees to actively encourage college students to innovate and start businesses (Lei and Jia, 2021). “2021 Shaanxi University graduates employment quality report” shows that in 2021, there were 333,228 college graduates in the province, an increase of 0.2 thousand to 2020, the initial employment rate of college graduates in the province was 86.98% (Shaanxi. Gov. Cn, 2022). The employment rate overall looks good; however, Shaanxi Province also faces the challenges of tight resources, insufficient policy support, and urgent job requirements for new graduates. Moreover, a factor ignored in the employment data is that in the current oversupply labor market of graduates, many people are forced to take jobs that do not require tertiary qualifications and do not use graduate skills, which means that the employment rates shown in the data may not be graduates’ true employment (Blenkinsopp and Scurry, 2007), furthermore, graduate employment data generally measures current employment status, rather than longer-term employability (Harvey, 2001). Thus, except for the employment policies when graduates move to the job market, training their employability from university enrollment is a great way to help them adapt to the labor market and increase the employment rate. Based on this background, we put forward three research questions:

Q1. What factors influence the improvement of graduates’ employability?

Q2. What factors are involved in employability?

Q3. What is the most important employability factor for students?

With these research questions, this article uses the quantitative analysis method to make research design and collect data, by analyzing the graduate’s employability in Shaanxi Province, exploring the factors which influence the improvement of the graduates’ employability as we as the systematic factors that employability involves, then rank these factors to find the most important ones, finally obtained research results. The findings of this article will help promote the graduates to become distinctive in terms of employability and better access to the labor market, also give colleges and universities a systematic training guide, reform graduates’ training mode, and provide beneficial references for China’s higher education personnel training.

## Literature review and research hypothesis

### Employability study

For the past few years, the study on employability has been frequently discussed (Cheng et al., 2021). In 1909, British economist Willam Beveridge first proposed the concept of employability, it can also be called employment ability, core ability, key competence, or employment skills (Huang et al., 2022). Sheffield Hallam University defines the employability of college students from the perspective of colleges and universities. They believe that the courses students have learned, the basic knowledge they have mastered, the cultivation they need in work, and the real self-realization through various incentive methods are their employability (Belbin, 1981). The representative view is that employability is a person’s ability to obtain a job, obtain employment, maintain employment, and re-employment, including skills, knowledge, and experience (Harvey, 2001). In addition, many policy studies define college students’ employability as a core vocational skill (Perez et al., 2010).

Law (1999) carried out the study on the components of employability earlier and developed a DOTS conceptual model of employability, which includes four elements considered basic elements of vocational education: decision-making learning, opportunity awareness, transition learning, and self-awareness. Bowe (1998) conducted a comprehensive study, and he proposed a relatively comprehensive and complete employability structure, including four parts: Assets, development, expression, and adaptation. Zinser (2003) thought that excellent college students and individuals should have the employment structure factors, including professional knowledge and professional skills, information searching ability, logic, response to emergencies, execution and team cooperation ability, effective expression and communication ability, career management and negotiation ability. Denisi (2004) studied and elaborated on the set of abilities that an excellent organization member should possess from the perspective of organization management, including results and actions, dedication and service awareness, influence, leadership, cognitive ability, and personal qualities. According to Li (2012), the employability of college students includes professional ability, interpersonal influence, analytical thinking, professional identity, and personal morality. Wang (2014) believed that the employability of college students refers to the various learning abilities and skills of the employed personnel according to the needs of the job. Hillage and Pollard considered employability as the ability needed for initial employment, they proposed maintaining the existing employment status and obtaining new employment and proposed four main components of employability: Employability assets, allocation, presentation, and an individual’s operating space (Hillage and Pollard, 1998; Llinares-Insa et al., 2018). Clarke (2018) proposed that employability includes six key dimensions, human capital, social capital, personal attributes, personal behavior, perceived employability, and labor market.

The literature study mentioned in previous paragraphs shows that there is a lack of agreement about what scales may assess college employability, and what specific factors are in assessing college employability (De Witte, 2005; Tymon, 2013). Thus, we summarized the college employability factors from different aspects to fill this gap. We think college graduates’ employability factors should include: Self-management ability, knowledge understanding and learning ability, career planning ability, professional ability, generic skills, and emotional skills.

### Individual traits and employability

**FIGURE 2:**
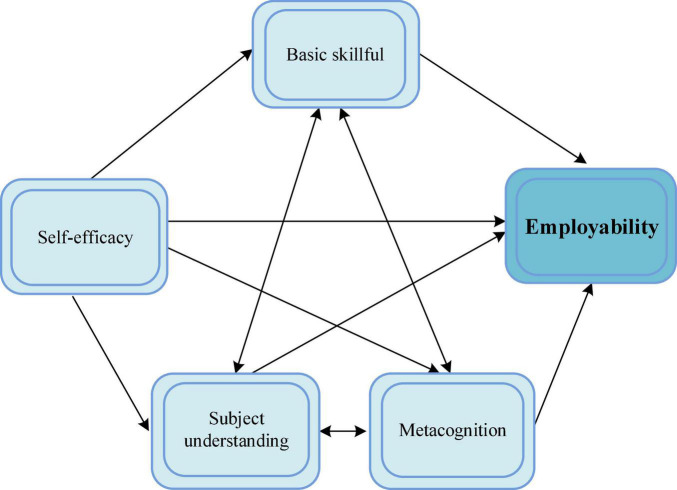
The USEM model.

Some scholars studied the personal influence on employability. British scholars jointly proposed the theoretical model of USEM (Yorke and Knight, 2004). The USEM model has four important dimensions, including subject understanding, basic skills, self-efficacy, and metacognition, as shown in Figure 2. Each of the dimension does not exist in isolation but influence each other. Skills refer to the abilities possessed and are divided into hard and soft skills (Zhang et al., 2022). Soft skills are universally applicable, while hard skills are unique and professional. Subject comprehension refers to the ability to be familiar with specific subject knowledge and to use it flexibly. Self-efficacy refers to having a rational and objective understanding and analysis of oneself. It also refers to maintaining a high degree of confidence and accurately judging one’s abilities in various aspects. So as to help the graduates to choose a suitable career more accurately. Metacognition is the assessment and understanding of one’s own conditions and social environment. It fully shows one’s initiative and ambition by continuously adjusting themselves during work time (Kenny et al., 2007).

Kearns (2001) believes that employability is composed of knowledge, skills, and attitudes focused on individuals, and is the synthesis of these three. Fugate et al. (2004) pointed out that employability is people-oriented, it is the subjective change and development of individuals. According to Tomlinson (2007), employability is the qualification or competitive ability that an individual has to adapt to the changing needs of employers or customers, and the ability to unleash his passion and potential in his study. In addition, in practice the focus also with a heavy emphasis on personal skills and ability (Bennett et al., 1999).

Most scholars focus on personal abilities when studying the personal influence on employability, but the role of individual attributes has largely been ignored (Fugate and Ashforth, 2003). We believe that personal attributes make an important role in the process of graduates finding jobs and succeeding in their chosen careers, thus, we put forward the following hypotheses.

Hypotheses 1: Individual traits have a positive impact on College students’ employability.

### Social experience and employability

Except for the individual aspect, some scholars’ analysis of factors that influence employability is from the perspective of the social side. Rajan et al. (2000) believed that the components of employability should be considered from both internal and external perspectives. The internal dimension refers to individual abilities and characteristics, while the external dimension refers to other than individual characteristics, such as macro labor market environment, industry conditions, and policy preference, which affect employment results to a certain extent (Rajan et al., 2000). From the perspective of social psychology, Mcardle et al. (2007) and Fantinelli et al. (2022) believed that employability should include personal adaptation, career cognition, human and social capital, and other factors. Andrew divided “perceived employability” into the perception of external factors such as labor market conditions, university reputation, and professional fields, and the perception of internal factors such as individual skill mix and workers’ self-belief (Rothwell et al., 2008). Holmes pointed out that social background has a significant direct effect on any indicator of employability (Holmes, 2013). Forret and Dougherty (2004) believed social capital has the potential to significantly improve graduate employability outcomes, social capital refers to the interpersonal relationship that an individual has, maintaining a good interpersonal relationship with others and being good at communication can help employees gain social recognition and actively seize opportunities for career development. Lu et al. (2017) confirmed that professional quality and social adaptability had a strong positive relationship with college students’ employability.

Summarized from the aforementioned literature, we find that when discussing the external influencing factors of employability, most scholars mainly focus on social factors, they have discussed social skills, social capital, and social environment but few of them have discussed the social experience. Thus, we put forward the following hypotheses.

Hypothesis 2: Social experience has a positive impact on College students’ employability.

### Workplace training and employability

Research proved that the employability of college students not only comes from college students themselves but is also shaped by the job market (Rothwell and Arnold, 2007).

**FIGURE 3:**
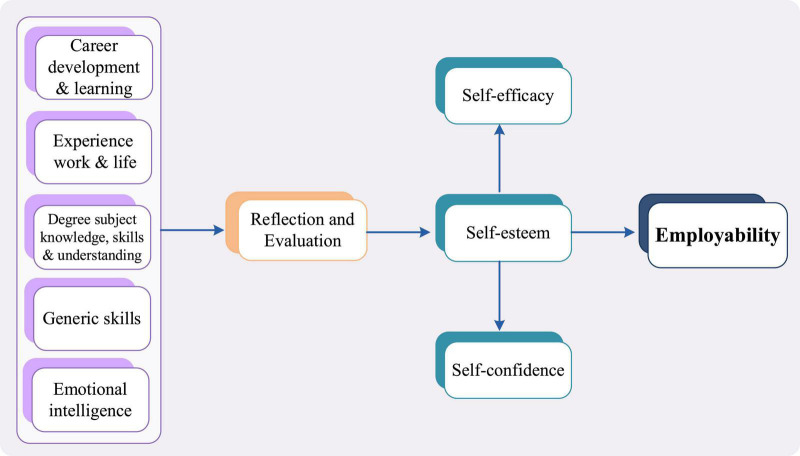
The career EDGE model.

British scholars Pool and Well proposed a new employability model—the Career EDGE model. This model stated that students should have the ability in five aspects: Career development learning, experience (work and life), degree subject knowledge understanding and skills, generic skills, and emotional intelligence (Pool and Sewell, 2007), as shown in Figure 3. This model shows that through career development learning, students will become more self-aware and able to fully recognize their career interests. Learning from work and life experiences, students’ employability can be improved. Professional knowledge and skills mainly refer to the understanding of applied discipline knowledge, professional background, etc. Generic skills can support people from any academic background and can be transferred and applied in a variety of settings (whether in school or the workplace). Emotional intelligence refers to a person’s quality emotions, will, tolerance to setbacks and so on, it is positively related to one’s achievements (Pitan and Atiku, 2017). Career EDGE model believes only with these abilities can development and progress be possible. Then, through self-reflection and evaluation, employees can evaluate and improve themselves. It can gradually develop into self-efficacy and self-confidence, which are important manifestations of self-esteem (Ayala Calvo and Manzano García, 2021). Therefore, the interrelation and interaction between these elements contribute to the framework of employability. Career EDGE model provides a complete framework of employability structure, from professional skills to quality experience, from external pressure to internal motivation, which all tell us what elements should be fully considered when conducting employability research (Krouwel et al., 2019; Strindlund et al., 2019). Career EDGE is considered the key model to analyze employability (Anderson and Tomlinson, 2021).

Positive career behavior is considered a key factor in building and enhancing employability (Fugate et al., 2004). Okay-Somerville and Scholarios (2017) believed that career self-management and associated behaviors such as career exploration, guidance seeking, and networking, are one of the main factors that promote employability. The best place to put the Career EDGE model and these behaviors into practice is in the workplace. Practice and challenge often stimulate and improve people’s ability, in order to obtain stronger employability, it is necessary to continue to experience and accumulate experience in the workplace, after workplace training, students will have a different experience from other college graduates. Thus, we put forward the following hypotheses.

Hypothesis 3: Workplace training has a positive impact on College students’ employability.

**FIGURE 4:**
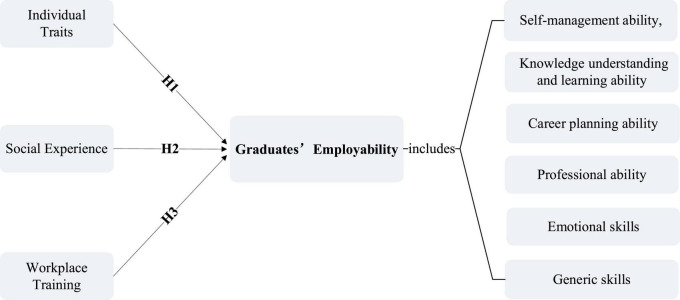
Research model.

This research model focuses on the aspects of individual traits, social experience, and workplace training of the current college graduates’ employability, and the college graduates’ employability consists of six elements: Self-management ability, knowledge understanding and learning ability, career planning ability, professional ability, generic skills, and emotional skills, shown in Figure 4.

## Methodology

The research on the evaluation of college students’ employment ability is mainly from the aspects of employment-related ability, it is necessary to use quantitative methods. The quantitative analysis method is the main methodology in this research, mainly reflected in the questionnaire survey. This article mainly uses the SPSS software to conduct reliability and validity analysis, principal component analysis, exploratory factor analysis, and multiple linear regression analysis on the questionnaire survey data based on the questionnaire survey, and finally reported the research findings.

### Questionnaire design

Before the compilation of the questionnaire, the survey between projects was carried out first through the open questionnaire quantity. On the basis of clarifying the connotation of college students’ employability, through the open questionnaire survey and literature review, and theoretical analysis, the general items of college students’ employability are considered. Then, the research used “*National Employment Survey of College Graduates”* questionnaire surveys in 2019 conducted by the *Economics of Education at Peking University* (Yue and Zhou, 2019) for reference, referred to relevant scientific research achievements and comprehensive literature, summarized the relevant influencing factors of college students’ employability from the literature we used above, and extracted 38 questions of employability according to the importance and correlation of the influencing factors, named “Survey on employability of college students.” The questionnaire contains five sections including the basic information of college graduates, current status, social practice experience, employment status, and the 38-items self-report question scale design, shown in Table 1. To measure the scale of the relativity of each dimension of employability, the Likert scale is used for each question in the questionnaire. A Likert scale typically offers five possible responses corresponding to different levels to a statement or question, allowing respondents to express their level of agreement or feeling about the issue or statement on a positive-to-negative scale (Göb et al., 2007). In such a five-tier rating system, a score of 5 means full compliance, a score of 4 means a good match, a score of 3 means uncertainty, a score of 2 means a bad match, and a score of 1 means a non-conformance. All questions are positive questions, which means the higher score, the higher the college graduates’ employability. On the contrary, the lower the score, the weaker the graduate’s employability.

**TABLE 1:** Survey on employability of college students.

Items	Questions	1	2	3	4	5
1	I can get the main point from all kinds of the information.					
2	I can analyze the information very fast and correctly.					
3	I can listen attentively and grasp the key information.					
4	I always have the clear logical analysis on solving problems.					
5	I am proficient in Office software and other software required for my work.					
6	I enjoy taking part in on-campus activities.					
7	I can prioritize and deal with multiple problems.					
8	I am good at observing the current situation and identifying problems.					
9	I will arrange my study and work time reasonably.					
10	I will not give up easily and I am responsible for my work.					
11	I will express myself at the right place and time.					
12	I will look for jobs through friends who are already employed.					
13	I will show my enthusiasm and energy in the process of job hunting.					
14	I can recognize my strengths and weaknesses and accept myself.					
15	I can make others feel comfortable when I get along with them.					
16	I often have a plan before doing something.					
17	I know how to express my strength to employers.					
18	I can communicate with others very well and express my opinion clearly.					
19	I have more than one solution to the same question.					
20	I can make decisions decisively.					
21	I can quickly adapt to different working environments and jobs.					
22	I often think differently about a problem than others.					
23	I like to experiment with different theories.					
24	I am willing to negotiate with others when I encounter problems.					
25	I often come up with ideas that others didn’t think of.					
26	I give priority to the interests of the group.					
27	I feel comfortable when working with my teammates.					
28 29	I feel comfortable when I am in charge of my team. I started to think about my future career when I was in the university.					
30	I know what kind of job I want.					
31	I am actively seeking promotions.					
32	I have clear stage goals and career planning.					
33	I know the long-term goal I am seeking in my career.					
34	People seek help from me for my professional knowledge.					
35	I ranked high in my academics in college.					
36	I think professional knowledge is very important for my career.					
37	I have a solid grasp of professional knowledge and skills					
38	My professional skills can meet the needs of the company.					

Questionnaires were distributed through “*Wen Juan Xing*,” which is a platform that can design and collect the questionnaire, the questionnaire link can be easily opened using any social media app commonly seen in China and thus can be easily spread *via* social media. We use paid methods in the investigation process to motivate the respondents’ participation, increase the enthusiasm for filling in the questionnaire and expand the scope of the questionnaire dissemination, everyone who finishes the questionnaire can get a lucky draw, and has a chance to get a cash bonus. The probability of reward is based on the number of answers and the number of prizes randomly drawn; the probability is random. The rewards will be attached to the system of the “*Wen Juan Xing*,” and those who complete the questionnaire will receive the rewards directly from the “*Wen Juan Xing*.”

### Sampling techniques and participants

The investigation date was from 1 to 30 June 2022. We used the resources of the college and distributed electronic questionnaires to different colleges, such as Shangluo University, Baoji University of Arts and Sciences, Xi’an University of Finance and Economics, and Shaanxi University of Science and Technology. The survey subjects comprised college students and graduates, as well as students of the given year who are already working. The questionnaires were distributed to each sampled institution of higher education at a certain ratio, we totally distributed 400 questionnaires to four universities, and after 4 weeks of data collection, excluding 180 invalid questionnaires with missing values or irregularities, 220 valid questionnaires were received.

In this research, the participants included 220 students and students who graduated from these four institutions. The gender distribution of this sample was 60% women and 40% men, 23% of the respondents were studying science, and 72% were studying liberal arts. In addition, 32% of the respondents were current students, 23% were students with social practice and 45% were already at work, as shown in Table 2.

**TABLE 2:** Descriptive statistics of the sample.

	Items	Quantity	Proportion (%)
Gender	Male	88	40
	Female	132	60
Major	Science	51	23
	Liberal arts	158	72
	Other	11	5
Current status	Student	70	32
	Student with social practice	50	23
	Employee	100	45
Total		220	100

## Data analysis and results

### Reliability and validity analysis

Before the analysis, this questionnaire uses Cronbach α value, a classic indicator often used in empirical research to test the reliability and accuracy of the answers to quantitative data. The statistician Hair pointed out that a Cronbach α score greater than 0.7 implies that the data is more credible (Perreault, 2011). The α coefficient is analyzed by SPSSAU. The test results are shown in Table 3. As it can be seen that the reliability coefficient value is 0.9, greater than 0.7, indicating that the reliability of the research data is good.

**TABLE 3:** The table of cronbach alpha.

Cronbach alpha		
N of items	N of questionnaires	Cronbach α
38	220	0.972

In addition, the research also calculated the correlation values between each item index and its dimension to further illustrate the content validity of the scale. As shown in Table 4, content validity analysis was conducted for a total of 38 analysis items. Factor loading value displays the correlation between factors (latent variables) and analysis items (Manifest variables). Standard load factor (std. Estimate) values are usually used to represent the correlation between factors and analysis items. If the standard load coefficient value is greater than 0.7, there is a strong correlation (Jennrich and Bentler, 2011). As the table shows, they are generally greater than 0.7. In general, items below 0.4 are considered for removal. Items 15,21,29,6, and 7 are during the value of 0.629–0.692, they are less than 0.7 but greater than 0.4. Given the small sample size, this may be the case. It was decided not to remove these items here. In terms of the measurement relationship, the absolute value of the standardized load coefficient is greater than 0.6 in each measurement relationship and presents significance, which means that there is a good measurement relationship. This indicates that the scale has good content validity.

**TABLE 4:** The correlation between factors and analysis items.

Items	Coef.	Std. error	Z	*p*	Std. estimate
1	1.000	−	−	–	0.733
2	0.983	0.089	11.09	0.000	0.78
3	1.053	0.094	11.22	0.000	0.789
4	1.013	0.092	11.05	0.000	0.777
5	1.000	−	−	–	0.725
6	0.971	0.1	9.672	0.000	0.684
7	0.887	0.091	9.794	0.000	0.692
8	1.165	0.096	12.12	0.000	0.864
9	1.000	−	−	–	0.738
10	1.027	0.089	11.59	0.000	0.768
11	0.956	0.088	10.84	0.000	0.722
12	1.009	0.089	11.32	0.000	0.752
13	0.976	0.087	11.24	0.000	0.746
14	1.078	0.088	12.3	0.000	0.811
15	0.924	0.099	9.34	0.000	0.629
16	1.068	0.093	11.48	0.000	0.761
17	1.041	0.086	12.05	0.000	0.796
18	0.999	0.086	11.59	0.000	0.768
19	1.000	−	−	–	0.784
20	0.969	0.081	11.99	0.000	0.749
21	0.897	0.085	10.59	0.000	0.676
22	1.156	0.085	13.61	0.000	0.827
23	0.981	0.083	11.88	0.000	0.743
24	1.000	−	−	–	0.866
25	1.140	0.087	13.04	0.000	0.8
26	1.074	0.083	12.87	0.000	0.792
27	0.899	0.061	14.78	0.000	0.811
28	0.924	0.06	15.44	0.000	0.833
29	1.000	−	−	–	0.668
30	1.097	0.112	9.777	0.000	0.736
31	1.227	0.114	10.75	0.000	0.823
32	1.228	0.111	11.04	0.000	0.85
33	1.196	0.114	10.52	0.000	0.802
34	1.000	−	−	–	0.769
35	0.969	0.083	11.63	0.000	0.757
36	0.965	0.084	11.42	0.000	0.745
37	0.981	0.079	12.43	0.000	0.801
38	0.970	0.08	12.07	0.000	0.781

This means that the internal consistency of the whole scale is satisfactory. Hence, the reliability and validity of the scale and sample are good and can be used for further analysis.

### Factor analysis

#### Indicators correlation test

Before doing factor analysis, the correlation between indicators should be tested. Kaiser–Meyer–Olkin (KMO) and Bartlett’s sphericity test are the measures to test the correlation of sample data. The larger the KMO value, the higher the contribution rate of the factor, and the more common factors between variables, the more suitable for factor analysis; if the KMO value is less than 0.5, it is not suitable for factor analysis. The Bartlett sphericity test is to test whether the correlation matrix is a unit matrix, that is, whether each variable is independent or not, whether the correlation coefficients between variables are related. If the significance probability of the chi-square statistic value of the Bartlett sphericity test is less than 0.01, it means that the data are correlated and are suitable for factor analysis (Basto and Pereira, 2012).

Table 5 shows the KMO and Bartlett sphericity test of the sample. It can be seen from the table that the KMO value is 0.953, higher than 0.5, which means that data can be used for factor analysis research. Bartlett sphericity test sig. = 0.000, less than 0.01, which means that there is a strong correlation between the research items and the factors, and the factors can effectively extract information.

**TABLE 5:** Kaiser–Meyer–Olkin (KMO) and Bartlett test.

KMO and Bartlett test		
KMO		0.914
Bartlett test	Approx. Chi-Square.	3224.754
	df	703
	Sig.	0.000

#### Extract common factors

**FIGURE 5:**
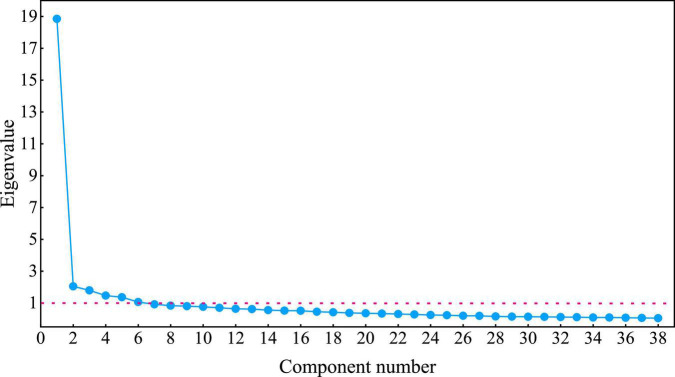
Scree plot.

In questionnaire data analysis, principal component analysis was carried out to extract common factors, and the scree plot in the principal component analysis was used for verification. The number of factors is determined by observing the eigenvalues of each factor in the data sample, and then the principal component analysis method is used for factor analysis. According to the Kaiser criterion, in factor analysis, only the factors whose eigenvalues are greater than 1 are retained. The common factors are selected according to the criterion that the cumulative contribution rate of the factors is greater than 60%, and the number of factors is determined based on the criterion that the eigenvalue is greater than 1 (Liu et al., 2003). Figure 5 is the Scree plot, reflecting the change in the eigenvalue. Table 6 reflects the variance contribution rate and the cumulative contribution rate of each factor.

**TABLE 6:** Total variance explained.

Number	Eigenvaluesl			Rotation sums of squared loadings		
	Total	% of variance	Cumulative %	Total	% of variance	Cumulative %
1	18.848	49.599	49.599	18.848	49.599	49.599
2	2.051	5.397	54.996	2.051	5.397	54.996
3	1.796	4.726	59.722	1.796	4.726	59.722
4	1.466	3.859	63.581	1.466	3.859	63.581
5	1.364	3.589	67.170	1.364	3.589	67.170
6	1.063	2.796	69.966	1.063	2.796	69.966
7	0.917	2.412	72.378	–	–	–
8	0.836	2.201	74.579	–	–	–
9	0.799	2.102	76.681	–	–	–
10	0.757	1.992	78.673	–	–	–
11	0.700	1.841	80.514	–	–	–
12	0.636	1.674	82.188	–	–	–
13	0.615	1.618	83.806	–	–	–
14	0.558	1.469	85.275	–	–	–
15	0.521	1.370	86.646	–	–	–
16	0.515	1.356	88.002	–	–	–
17	0.454	1.196	89.197	–	–	–
18	0.421	1.109	90.306	–	–	–
19	0.380	0.999	91.305	–	–	–
20	0.358	0.942	92.247	–	–	–
21	0.334	0.878	93.125	–	–	–
22	0.306	0.806	93.931	–	–	–
23	0.282	0.741	94.673	–	–	–
24	0.248	0.652	95.324	–	–	–
25	0.235	0.618	95.943	–	–	–
26	0.201	0.530	96.473	–	–	–
27	0.190	0.501	96.974	–	–	–
28	0.155	0.409	97.383	–	–	–
29	0.145	0.382	97.765	–	–	–
30	0.135	0.355	98.120	–	–	–
31	0.127	0.335	98.455	–	–	–
32	0.114	0.300	98.754	–	–	–
33	0.109	0.286	99.040	–	–	–
34	0.089	0.235	99.275	–	–	–
35	0.086	0.226	99.501	–	–	–
36	0.075	0.197	99.698	–	–	–
37	0.065	0.171	99.869	–	–	–
38	0.050	0.131	100.000	–	–	–

Extraction method: Principal component analysis.

From the Scree plot, it can be seen that there have six factors whose eigenvalues are greater than 1, the slope of all data factors is flattened from the seventh factor, so it is more appropriate to keep six factors. The data in Table 6 clearly shows that the cumulative variance contribution rate of the first six factors reaches 69.966%, which is greater than the preset 60% standard, is a high degree of explanation, indicating that these six factors can broadly include the variable information in the original test data, and the graduate employability could be condensed into six clusters with these eigenvalues.

In conclusion, it can be seen that the 38 items in the employability structure of college students in the factor analysis have been well explained, indicating that the extracted six common factors can make their meanings clearer, the cluster analysis is clearer, and can provide a good data basis.

#### Factor matrix analysis

The factor loading matrix is the coefficient of each factor expression in the original variable, which reflects the degree of influence of the extracted common factor on the original variable. According to the characteristic that the coefficient correlation of the initial factor loading matrix is not significant in factor analysis (Belohlavek and Krmelova, 2014), this article uses the maximum variance method to orthogonally rotate the factor loading matrix of the test data, and re-rotate the correlation between the six factors and the original variable, so that the representativeness of common factors is more in line with the actual situation and can better explain the correlation between the factors. The factor loading matrix after rotation is shown in Table 7. According to the result of the factor loading rotation matrix, each project has a large load in the corresponding factor, ranging from 0.407 to 0.756, indicating that each factor is closely related to the corresponding original factor, and the degree of dependence on the factor is relatively high.

**TABLE 7:** Rotated component matrix.

Items	Components					
	Factor 1 knowledge understanding and learning ability	Factor 2 professional ability	Factor 3 emotional intelligence	Factor 4 self-management ability	Factor 5 career planning	Factor 6 generic skills
1	0.756					
2	0.739					
3	0.687					
4	0.521					
5						0.581
6						0.689
7						0.601
8						0.603
9				0.545		
10				0.584		
11				0.607		
12				0.572		
13				0.625		
14				0.610		
15				0.729		
16				0.655		
17				0.590		
18				0.488		
19			0.630			
20			0.532			
21			0.672			
22			0.659			
23			0.702			
24			0.585			
25			0.447			
26			0.565			
27				0.669		
28				0.696		
29					0.653	
30					0.704	
31					0.543	
32					0.53	
33					0.591	
34		0.584				
35		0.665				
36		0.609				
37		0.723				
38		0.662				
Eigenvalues	6.53	5.239	5.025	4.208	4.021	1.565
% Of variance	17.184%	13.786%	13.223%	11.073%	10.581%	4.118%
Cumulative %	17.184%	30.970%	44.193%	55.266%	65.847%	69.965%

Extraction method: Principal component analysis. Rotation method: Varimax with Kaiser Normalization.

Rotation converged in 3 iterations.

The research results are further normalized, after rotating, naming each factor according to the rotated factor component matrix, and calculating the weight coefficient (K) by each factor’s eigenvalue (λ).

Factor 1 contains five items, and includes questions 1–4, and 27–28, which are knowledge understanding, and learning ability. It is about knowledge summary, memory, expression, comprehension, and application. The weight corresponding to factor 1 is set to *K*_1_ = λ_1_/(λ_1_ + λ_2_ + λ_3_ + λ_4_ + λ_5_ + λ_6_) = 24.56%.

Factor 2 contains five items, namely questions 34–38, which is professional ability. Which measures the knowledge and capability in a professional field, including professional knowledge, skills, ability, quality, style, and spirit. The weight corresponding to factor 2 is set to *K*_2_ = λ_2_/(λ_1_ + λ_2_ + λ_3_ + λ_4_ + λ_5_ + λ_6_) = 19.7%.

Factor 3 contains seven questions, questions 19–26, named emotional skills. It includes self-awareness, emotional control, self-motivation, recognizing the emotions of others, and processing relationships. The weight corresponding to factor 3 is set to *K*_3_ = λ_3_/(λ_1_ + λ_2_ + λ_3_ + λ_4_ + λ_5_ + λ_6_) = 18.9%.

Factor 4 contains 10 items, including questions 9–18, named self-management ability. Refers to strategies that use one’s inner strength to change behavior, focusing on one’s self-teaching and restraint power, such as time management, goal management, interpersonal management, stress management, behavior management, emotion management, responsibility, and the sense of self-discipline. The weight corresponding to factor 4 is set to *K*_4_ = λ_4_/(λ_1_ + λ_2_ + λ_3_ + λ_4_ + λ_5_ + λ_6_) = 15.83%.

Factor 5 is career planning ability, including questions 29–33. It means that employees have an in-depth understanding of their own abilities, make a long-term plan for their future, have a program to get promoted and improve themselves, and have career interests, career goals, career abilities, promotion plans, career orientation, and career development. The weight corresponding to factor 5 is set to *K*_5_ = λ_5_/(λ_1_ + λ_2_ + λ_3_ + λ_4_ + λ_5_ + λ_6_) = 15.12%.

Factor 6 is generic skills and includes topics 5–8. Which measures by problems solving ability and proficiency in working. The weight corresponding to factor 6 is set to *K*_6_ = λ_6_/(λ_1_ + λ_2_ + λ_3_ + λ_4_ + λ_5_ + λ_6_) = 5.89%.

According to the results of the abovementioned exploratory factor analysis method, the structure of college students’ employability is a structured model including 38 indicators in six dimensions. Through the weight coefficient of each factor, it is clear that knowledge understanding and learning ability have the greatest impact on the employability structure, emotional intelligence, professional ability, self-management ability, and career planning ability are the followed factors, at an average weight level, the generic ability is the last factor, has the lowest weight.

### Multiple linear regression analysis

For exploring the relationship between individual characteristics, social experience, workplace training, and employability, and verifying the hypothesis, we now use multiple linear regression analysis. Multiple linear regression is a statistical analysis method that uses regression analysis in mathematical statistics to determine the quantitative relationship between two or more variables, it aims to model the linear relationship between the independent variables and the dependent variables (Morrow-Howell, 1994). After factor analysis, we confirmed six factors of employability, according to the literature summary and our survey experience, we distributed these six factors into individual characteristics, social experience, and workplace training aspects. Self-management ability, knowledge understanding, and learning ability are grouped into individual characteristics, generic skills, emotional skills are grouped into social experience, career planning ability, and professional ability are grouped into workplace training. Thus, we are going to measure the hypothesis by testing these six factors.

Table 8 is the multiple linear regression results of the model. From the table, we can see that VIF < 10, which means that the collinearity among explanatory variables is weak after the multiple collinearity test. The *t*-value is the result of the *t*-test for the regression coefficient. The larger the absolute value is, the smaller the Sig is. Sig represents the significance of the *t*-test. The Sig. of the regression model is < 0.01, which indicates that there is a highly significant linear relationship between explanatory variables and explained variables. β of these six factors is all positive, indicating that six factors have a significant positive effect on college graduates’ employability, which also means that individual characteristics, social experience, and workplace training aspects have a positive influence on employability.

**TABLE 8:** Multiple linear regression.

	Unstandardized coefficients		Standardized coefficients	t	Sig.	VIF
Variables	B	Std. error	B			
Self-management ability	7.111	0.948	0.523	7.503	0.00	6.916
Knowledge understanding and learning ability	6.725	0.636	0.9	10.567	0.00	5.426
Generic skills	6.648	0.989	0.448	6.722	0.00	7.784
Emotional skills	9.288	1.265	0.751	7.345	0.00	9.537
Career planning ability	6.115	0.817	0.521	7.489	0.00	6.919
Professional ability	5.781	0.786	0.88	7.353	0.00	9.968

Dependent Variable: College employability.

**FIGURE 6:**
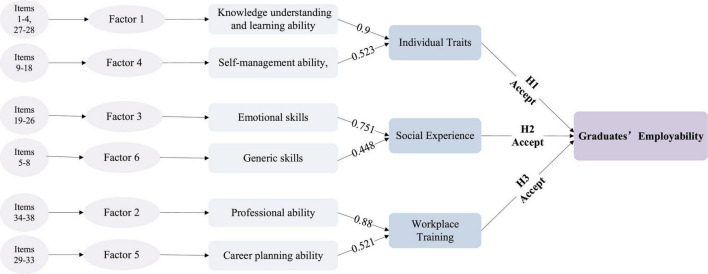
Final research model.

Data shows that there was a positive correlation between knowledge understanding and learning ability (β = 0.9, *t* = 10.567, *P* < 0.01) and self-management ability (β = 0.523, *t* = 7.503, *P* < 0.01) and college students’ employability. The more outstanding the understanding and learning ability of individual college students, the stronger their own employability; the stronger the self-management ability, the stronger their own employability, which indicates that individual character has a significant influence on employability. The coefficient of the emotional skills and generic skills on college students’ employability are both positive (β = 0.751, *t* = 7.345, β = 0.448, *t* = 6.722, *p* < 0.01), which indicates that social experience is also important for college students’ employability improvement, it is for sure that social experience has a strong effect on employability, students who have internship experience or other social activity experience with stronger employability. The coefficient of the professional ability (β = 0.880, *t* = 7.353, *p* < 0.01), and career planning ability (β = 0.521, *t* = 7.489, *p* < 0.01) are both positive, to prove that people with technic skills and occupational ideal has stronger employability indicating that workplace training has a positive effect on employability. This verifies that the H1, H2, and H3 are correct, and the final structure model is shown in Figure 6.

## Discussion

### Theoretical implications

Based on the college perspective, this study explores the factors influencing the enhancement of college graduates’ employability, and the findings have important theoretical implications. This research provides a new perspective for the study of employability and offers new research findings in this field, it is helpful to enrich the theories related to college graduates’ cultivation mode in colleges.

First, this research includes individual characteristics, social experience, and workplace training into the employability dimension, it breaks the previous literature that only considers the individual or the society when studying employability, and it makes the research on the influencing factors of employability more comprehensive and reasonable. Second, this article takes students from universities in Shaanxi Province as the research object, it is more specific than previous studies on national employability, and it will help deepen the analysis and research on the factors of college graduates’ employability in Shaanxi Province. Third, this study finds six specific dimensionalities of employability factors, showing that employability is a multidimensional meta-competence, rather than a one-dimensional structure (Cuyper et al., 2008), it extends previous research and updates new employability measurement dimensions, provides an excellent theoretical basis and proof for research in employability field, has great guiding significance to the future study of employability.

### Practical implications

The renewal of the understanding of employability would have a long-term practical impact on individuals, universities, and corporations.

Primally, this article summarizes an employability scale for students to measure their job competence, it tells students what kind of employability they should have. Therefore, college graduates can evaluate their own abilities scientifically, building their own ability structure and the new employment form of their own ability requirements, as well as put targeted efforts to improve their employability, which will help them better find employment, and enter the market more quickly. It has universal use value and practical guiding significance for enhancing the core competitiveness of college students in employment.

Moreover, institutions are the main place to cultivate students’ employability and play a vital role in cultivating and enhancing the employability of college graduates (Nicolescu and Nicolescu, 2019). This research provides a beneficial reference for China’s higher education personnel training and reform graduates’ training mode and cultivates practical graduates to meet the needs of society, it also offers policymakers and lecturers in universities the guideline to teach the students. Universities can use these research findings as guidance to help students gain stronger employability by changing the education mode, focusing on using college education, and providing students with opportunities to consolidate their abilities.

Most importantly, this research has important practical value for promoting sustainable and healthy development in the labor market. Different departments of the organization have different requirements for employees’ abilities. For example, sales staff need professional sales skills and high emotional intelligence, while technical departments need professional technology. College students can apply for corresponding departments according to the employability which they are good, thus reducing the pressure on enterprises for new employee training. Furthermore, when graduates enter the labor market with a clear career goal, they will not have incompatibility problems of the relevant occupational ability, thus they would not have a lot of turnover problems and reduce the labor market pressure.

### Limitations and future prospects

This study tries to obtain a better understanding of employability factors in the new situation, and it has some limitations.

First, as the quantitative method, this research used is based on the self-evaluation of the subjects on their own skill. It is difficult to keep people from being biased in evaluating themselves. This research has not identified the extent of bias people have on their own skills and has not adjusted the data based on bias. As a result, the data collected might differ if the research is done by peer review. In future research, more objective data can be used, or experiments can be carried out to explore.

Second, this thesis has only investigated one specific period and area, the number of subjects who participated in the quantitative research is 220, which is largely composed of people with direct or indirect personal relationships with the researcher. This likely created some bias in geographical location and common experiences of research subjects, how much the results are replicable throughout different parts of Shaanxi is unproven, and it is undiscovered how employability in the other region. The scope of data surveys can be expanded in future research to improve the reasonableness and representativeness of the sample and make the research results more convincing.

## Conclusion

This research takes the university graduates’ employability as the breakthrough point, and comprehensively analyzes the factors that influence the improvement of the employability of college students, as well as the factors that employability involved. This study uses SPSS software to conduct 220 valid questionnaires and verifies questionnaire quality through reliability and validity analysis. It uses factor analysis and multiple linear regression methods to expound the correlation between factors in the structure of college students’ employability, and finally obtains the research results which are three factors that influence employability improvement and the six factors that college graduates’ employability includes.

According to the research findings, individual characteristics, social experience, and workplace training have a significant positive impact on employability. The employability hierarchy factors of college students are as follows: Knowledge understanding and learning ability, professional ability, emotional intelligence, self-management ability, career planning ability, generic ability, knowledge understanding, and learning ability are the most important employability for college graduates.

## Data availability statement

The original contributions presented in this study are included in the article/supplementary material, further inquiries can be directed to the corresponding author.

## Author contributions

LJ performed conceptualization, data curation, formal analysis, visualization, writing—original draft, writing—review and editing, and resources. ZC performed investigation, formal analysis, software, formal analysis, project administration, and methodology. CL performed supervision, visualization, validation, and funding acquisition. All authors contributed to the article and approved the submitted version.
